# Associations of advanced age with comorbidity, stage and primary subsite as contributors to mortality from colorectal cancer

**DOI:** 10.3389/fpubh.2023.1101771

**Published:** 2023-04-06

**Authors:** Kazzem Gheybi, Elizabeth Buckley, Agnes Vitry, David Roder

**Affiliations:** ^1^University of South Australia Allied Health and Human Performance, Adelaide, SA, Australia; ^2^University of South Australia, Cancer Epidemiology and Population Health, Adelaide, SA, Australia; ^3^Charles Perkins Centre, School of Medical Sciences, University of Sydney, Sydney, NSW, Australia; ^4^University of South Australia Clinical and Health Sciences, Adelaide, SA, Australia

**Keywords:** advanced age, mortality, comorbidity, cancer stage, colorectal cancer

## Abstract

**Background:**

Although survival from colorectal cancer (CRC) has improved substantially in recent decades, people with advanced age still have a high likelihood of mortality from this disease. Nonetheless, few studies have investigated how cancer stage, subsite and comorbidities contribute collectively to poor prognosis of older people with CRC. Here, we decided to explore the association of age with mortality measures and how other variables influenced this association.

**Methods:**

Using linkage of several administrative datasets, we investigated the risk of death among CRC cases during 2003–2014. Different models were used to explore the association of age with mortality measures and how other variables influenced this association.

**Results:**

Our results indicated that people diagnosed at a young age and with lower comorbidity had a lower likelihood of all-cause and CRC-specific mortality. Aging had a greater association with mortality in early-stage CRC, and in rectal cancer, compared that seen with advanced-stage CRC and right colon cancer, respectively. Meanwhile, people with different levels of comorbidity were not significantly different in terms of their increased likelihood of mortality with advanced age. We also found that while most comorbidities were associated with all-cause mortality, only dementia [SHR = 1.43 (1.24–1.64)], Peptic ulcer disease [SHR = 1.12 (1.02–1.24)], kidney disease [SHR = 1.11 (1.04–1.20)] and liver disease [SHR = 1.65 (1.38–1.98)] were risk factors for CRC-specific mortality.

**Conclusion:**

This study showed that the positive association of advanced age with mortality in CRC depended on stage and subsite of the disease. We also found only a limited number of comorbidities to be associated with CRC-specific mortality. These novel findings implicate the need for more attention on factors that cause poor prognosis in older people.

## 1. Background

CRC is the second leading cause of cancer death in Australia, despite increases in 5-year relative survival in recent decades which now approximates 70% (1). Older age is still associated with higher case fatality, however, with 5-year survival now close to 60% in those who were aged 80 years or more at diagnosis. The number of older CRC patients is increasing markedly with increased population sizes in the older age brackets in Australia, along with their age-related comorbidity (2, 3).

Older age is found to be associated with poorer CRC outcomes, irrespective of the statistical methodology (i.e., all-cause/cancer-specific or short-term/long-term mortality) (4–6). This is so despite the fact that older people are often diagnosed at an earlier CRC stage than younger people (3, 7), with potential influences from increased comorbidity and frailty. While many studies reported lower survival among older patients, the literature is fairly sparse on the respective quantitative contributions of comorbidities, diagnostic stage, and cancer subsite in combination. We found only one study that had studied the impact of age on the CRC mortality by cancer stage (8).

Comorbidities are well-established predictors of CRC survival, but little evidence exists on the contribution made by specific comorbidities to CRC mortality. One study found that only some comorbidities impacted on CRC-specific mortality (5), although most comorbidities were associated with all-cause mortality (5, 9).

This study focusses on how stage, subsite and comorbidity influence the association of age with mortality in CRC patients. We also examine the association of individual comorbidities with CRC-specific and all-cause mortality.

## 2. Materials and methods

### 2.1. Study population and variables

People with colorectal cancer (C18-C20, ICD-10, International Classification of Disease) recorded by South Australian Cancer Registry (SACR) between January 1st, 2004–December 31st, 2013 were enrolled for this study. Pathology laboratories and health care centers in South Australia are mandated to notify SACR as a State-government registry of any malignancies diagnosed in their facilities (10). Through linkage with Deaths and Marriages (BDM) and the National Death Index (NDI) data, follow-up death data were obtained for the period up to December 31, 2014. Vital status and cause of death information were provided by the SACR using ICD-10 codes with a unique identification number for each patient. The minimum follow-up time in the study was 370 days. The underlying cause of death was derived from the death certificate issued by a certified medical practitioner and was based on the World Health Organization's rules for attribution of cause of death (10). This included death from CRC (disease specific) when recorded and verified by the SACR (C18–C20). Otherwise, death was classified as attributed to “another cause” (i.e., non-CRC) cause of death. The combination of the two indicated all-cause mortality. Socioeconomic status was categorized from most to least disadvantaged (Q1 to Q5) based on Index of Relative Socio-economic Disadvantage (11).

**Figure 1:**
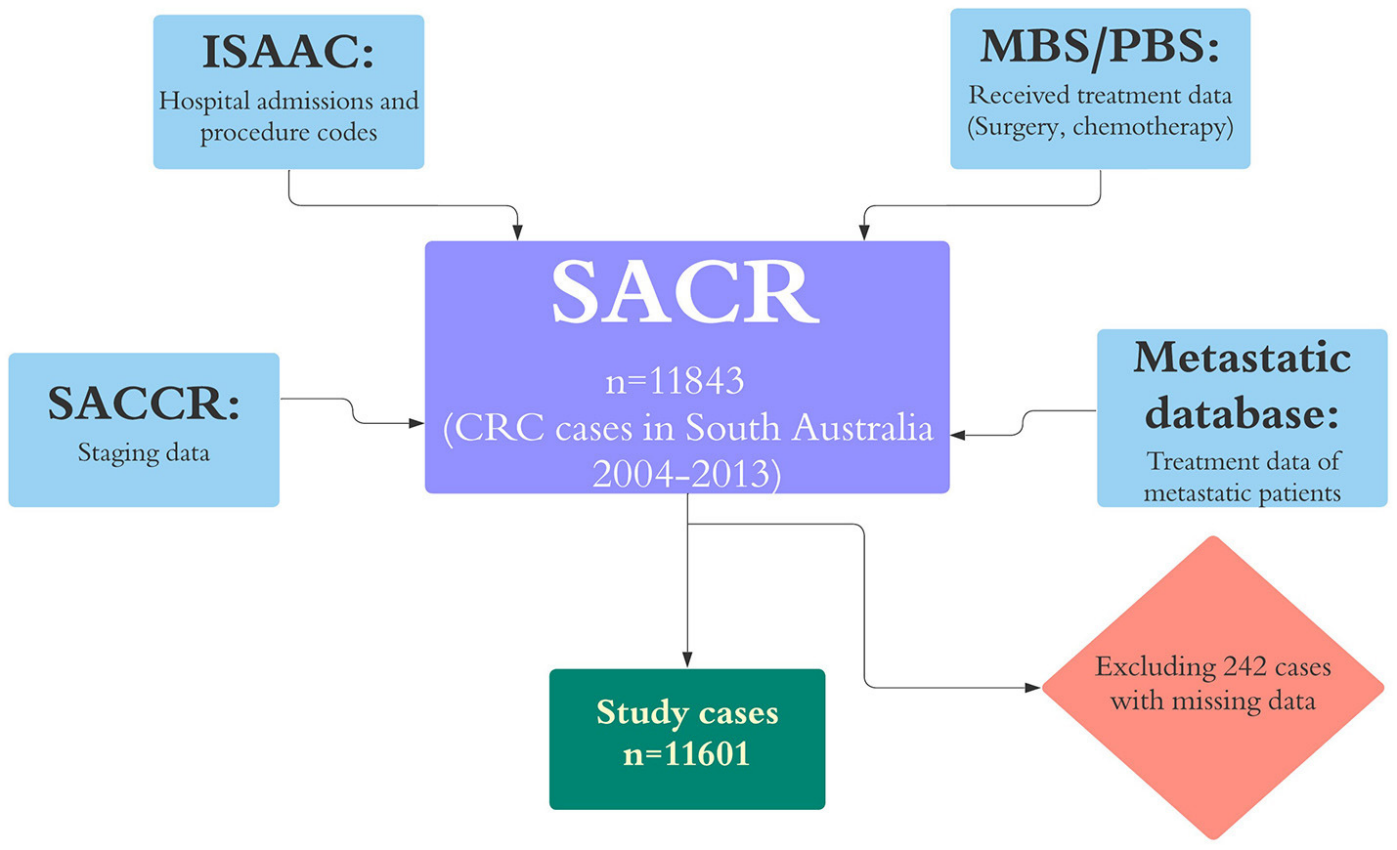
Datasets that were linked to SACR for this study and excluded cases. ISAAC, the Integrated South Australian Activity Collection; MBS, the Medicare Benefits Schedule; PBS, the Pharmaceutical Benefits Scheme; RAH, the Royal Adelaide Hospital; ARC, the Adelaide Radiotherapy Centre; and SACR, the South Australian Cancer Registry.

Cases with missing data on date of diagnosis and those whose diagnosis was changed (e.g., anal canal cancer) were excluded from the study (242 cases). The stage of CRC was provided by the South Australia Clinical Cancer Registry using Australian Clinico-Pathological Staging (ACPS), which is an extension of Duke staging (12). Where stage was unknown on the registry we categorized cases as advanced stage where so indicated by hospital admission records {i.e., stage C for metastasis to regional lymph nodes and stage D for more distant metastases (C77–C79) (13)}. Comorbidity burden was measured by Charlson comorbidity index (CCI) (14), using morbidities recorded in hospital inpatient data that were present on admission (15, 16). Treatment data were extracted from multiple sources and defined as binary variables (receipt of a treatment or not). The datasets that were used in this study and the linkage process is shown in Figure 1.

### 2.2. Statistical analysis

Survival from CRC-specific and all-cause mortality at one, two, five and ten years by age group was estimated by Kaplan-Meier analysis. All-cause mortality was also used as the outcome in Cox proportional hazards regression for multiple independent variables. We also used a competing risk model to investigate associations of different variables, including age, stage, comorbidity and treatment with CRC-specific mortality (17). This model accounted for deaths from non-CRC competing deaths, with results expressed as sub-distribution hazard ratios (SHRs). Time was estimated by days from CRC diagnosis date to the date of death or end of follow-up, whichever occurred first. The model was first adjusted by the variable described apart from treatment, and then with inclusion of treatment types. The purpose was to infer the influence of treatment on associations of age, comorbidity and stage with mortality. In all models, where testing for proportionality and, if this requirement not to be met, time-varying covariates were included (18–20). The models outcome were reported by Hazard ratio and SHR with a 95% confidence interval. *P*-values of <5% were considered as a statistically significant result.

The models estimated mortality by comorbidity status (CCI = 0, CCI = 1, 2 and CCI > 2), stage (stage A, B, C, D and unknown) and subsites (right and left colon, rectum) to explore whether variations in associations with mortality were indicated across these variables (by comparing the range of confidence intervals). In order to test if associations of age with mortality varied by subsite, stage and the level of comorbidity, interaction terms were tested. Among interaction term analyses for stage, stages A and B were combined due to small numbers of deaths.

Associations of individual comorbidities selected from the CCI index with CRC-specific mortality and all-cause mortality was also measured to assess whether these comorbidities showed different associations.

## 3. Results

### 3.1. CRC survivals based on age groups

Among the 11,601 CRC cases, 6233 (53.7%) were alive at the date censoring of December 31, 2014, 3,863 (33.3%) had experienced a CRC-specific death, and 1,505 (12.9%) had died of another cause. Table 1 shows descriptive features of the cases and their survival status at the end of the study period. Patients of older age, higher comorbidity, and advanced stage accounted for a high proportion of deaths.

**Table 1:** Characteristic of CRC cases and death outcomes by December 31, 2014: South Australia, 2004–2013 diagnoses.

**Variable**		**Total (%)**	**All-cause death (%)**	**CRC-specific death (%)**
Age group	≤50	922 (7.9)	306 (5.7)	271 (7.2)
(years)	51–60	1,741 (15.0)	624 (11.6)	566 (14.6)
	61–70	2,859 (24.6)	1,041 (19.4)	842 (21.8)
	71–80	3,495 (29.8)	1,647 (30.7)	1,130 (29.3)
	>80	2,620 (22.6)	1,750 (32.6)	1,054 (27.3)
CCI	0	6,107 (52.6)	2,224 (41.4)	1,823 (47.2)
	1, 2	3,560 (30.7)	1,803 (33.6)	1,262 (32.7)
	>2	1,934 (16.7)	1,341 (25.0)	778 (20.1)
Socioeconomic quintile^*^	Q1	2,885 (24.9)	1,397 (26.0)	1,022 (26.5)
	Q2	2,592 (22.3)	1,196 (22.3)	874 (22.6)
	Q3	2,215 (19.1)	1,027 (19.1)	699 (18.1)
	Q4	2,191 (18.9)	1,003 (18.7)	733 (19.0)
	Q5	1,716 (14.8)	744 (13.9)	534 (13.8)
Remoteness	Major cities	8,991 (77.5)	4,166 (77.6)	2,950 (76.4)
	Regional areas	2,270 (19.6)	1,027 (19.1)	780 (20.2)
	Remote areas	340 (2.9)	175 (3.3)	133 (3.4)
Primary subsite	Right colon	4,634 (39.9)	2,159 (40.2)	1,469 (38.0)
	Left colon	3,171 (27.3)	1,479 (27.6)	1,076 (27.9)
	Rectum	3,796 (32.7)	1,730 (32.2)	1,318 (34.1)
Sex*	Male	6,276 (54.9)	2,915 (54.6)	2,059 (5.6)
	Female	5,163 (45.1)	2,427 (45.4)	1,779 (46.4)
Stage	A	1,113 (9.6)	234 (4.4)	72 (1.9)
	B	1,947 (16.8)	648 (12.1)	323 (8.4)
	C	2,928 (25.2)	1,247 (23.2)	919 (23.8)
	D	2,475 (21.3)	1,990 (37.1)	1,800 (46.6)
	Unknown	3,138 (27.1)	1,249 (23.3)	749 (19.4)
Tumor differentiation (grade)	Well	581 (5.0)	113 (3.3)	113 (2.9)
	Moderate	7,629 (65.8)	3,086 (57.5)	2,036 (52.7)
	Poor/undifferentiated	2,153 (18.6)	1,233 (23.0)	983 (25.5)
	Unknown	1,238 (10.7)	869 (16.2)	731 (18.9)
Diagnostic period	2004–2008	5,861 (50.5)	3,315 (61.7)	2,287 (59.2)
	2009–2013	5,740 (49.5)	1,496 (38.2)	1,576 (40.8)

^*^12 and 162 cases had missing data in administrative datasets for socioeconomic status and sex respectively.

Table 2 shows the all-cause and CRC-specific survival functions by age group using Kaplan-Meier analyses. A comparison of the two survivor functions shows that CRC-specific survival was often close to all-cause survival for younger ages, and short follow-up intervals. However, for a 10-year interval, the difference between the two types of survival increases, especially for people older than 80 years. For example, for those aged 80+ years, the CRC-specific survival at 10 years was 0.41 (0.34–0.48) compared to 0.07 (0.04–0.10) for all-cause survival. Comparison of survival estimates and confidence intervals indicated that people younger than 70 years had similar CRC-specific survival within the study period. Meanwhile, people older than 70 years had lower survivals compared to younger age groups which was more evident after 5–10 years. Supplementary Figure S1 shows the Kaplan-Meier curves for all-cause and CRC-specific survivals. We can observe that the CRC-specific curves specially for cases older than 70 experience a plateau within the 5–10-year interval, however, this pattern is not seen for all-cause curves.

**Table 2:** Kaplan-Meier analyses for all-cause and CRC-specific survival by age groups: South Australia, 2004–2013 diagnoses, date of censoring: 31 December 2014.

**Variable**		**Survival (95% CI)**			
**Age groups (mean follow-up)**		**1 year**	**2 years**	**5 years**	**10 years**
≤50 years	AC	0.89 (0.87–0.91)	0.78 (0.75–0.81)	0.64 (0.60–0.67)	0.44 (0.35–0.52)
(1,364.0 days)	CS	0.90 (0.88–0.92)	0.80 (0.77–0.83)	0.67 (0.63–0.70)	0.48 (0.38–0.57)
51–60 years	AC	0.87 (0.86–0.89)	0.78 (0.76–0.80)	0.63 (0.61–0.66)	0.40 (0.34–0.45)
(1,403.5 days)	CS	0.88 (0.87–0.90)	0.80 (0.78–0.82)	0.66 (0.63–0.67)	0.44 (0.38–0.49)
61–70 years	AC	0.87 (0.86–0.88)	0.79 (0.77–0.80)	0.62 (0.60–0.64)	0.39 (0.35–0.44)
(1,382.4 days)	CS	0.89 (0.88–0.90)	0.81 (0.80–0.83)	0.67 (0.65–0.69)	0.51 (0.46–0.55)
71–80 years	AC	0.80 (0.79–0.82)	0.71 (0.69–0.72)	0.52 (0.50–0.54)	0.25 (0.21–0.30)
(1,283.3 days)	CS	0.84 (0.83–0.85)	0.76 (0.74–0.77)	0.64 (0.62–0.66)	0.47 (0.42–0.53)
>80 years	AC	0.64 (0.62–0.65)	0.52 (0.50–0.53)	0.30 (0.28–0.33)	0.07 (0.04–0.10)
(868.5 days)	CS	0.71 (0.69–0.73)	0.62 (0.60–0.64)	0.51 (0.49–0.54)	0.41 (0.34–0.48)

AC, All-cause survival; CS, CRC-specific survival.

### 3.2. Association of age and other clinicodemographic variable with mortality

The association of all-cause and CRC-specific mortality with study variables was measured (Table 3). Cancer stage was the strongest predictor of both all-cause and CRC-specific mortality (stage D all-cause mortality at HR = 12.33, 95% CI = 9.76–15.56, and CRC-specific mortality: SHR = 30.95, 95% CI = 20.23–47.35). Younger age was significantly associated with lower all-cause and CRC-specific mortality than those aged 80 years or over. This association was stronger for all-cause mortality than CRC-specific mortality (e.g., for the 71–80 age group, HR = 0.57, 95% CI = 0.53–0.61, and the SHR = 0.79, 95% CI = 0.72–0.88). The association of comorbidity was stronger with all-cause mortality than CRC-specific mortality (HR = 2.08, 95% CI = 1.94–2.24 vs. SHR = 1.20, 95% CI = 1.09–1.32). People with CCI > 2 had poorer all-cause mortality than people with CCI = 1, 2 (HR = 2.08, 95% CI = 1.94–2.24 vs. HR = 1.37, 95% CI = 1.29–1.46). Site of the cancer was not significantly associated with all-cause mortality, however, left colon and rectal cancers were significantly associated with higher CRC-specific mortality compared with right colon cancer.

**Table 3:** Associations of age, comorbidity status (CCI), stage (ACPS), grade, primary subsite, and treatment variables with all-cause (HR) and CRC-specific (SHR) mortality (multivariable regression): South Australia, 2004–2013 diagnose, date of censoring December 31, 2014^*^.

**Variable**		**All-cause mortality**		**CRC-specific mortality**	
**(Reference)**		**HR (95% CI)** ^+^	**HR (95% CI)** ^‡^	**SHR (95% CI)** ^†^	**SHR (95% CI)** ^‡^
Age group	≤50	**0.33 (0.29–0.37)**	**0.34 (0.30–0.39)**	**0.54 (0.47–0.62)**	**0.53 (0.45–0.61)**
(80 <)	51–60	**0.41 (0.37–0.45)**	**0.44 (0.39–0.48)**	**0.74 (0.66–0.83)**	**0.74 (0.65–0.84)**
	61–70	**0.43 (0.40–0.46)**	**0.47 (0.43–0.51)**	**0.72 (0.65–0.80)**	**0.73 (0.65–0.82)**
	71–80	**0.57 (0.53–0.61)**	**0.61 (0.57–0.66)**	**0.79 (0.72–0.87)**	**0.80 (0.72–0.88)**
CCI	CCI = 1, 2	**1.37 (1.29–1.46)**	**1.35 (1.27–1.44)**	**1.11 (1.04–1.20)**	**1.10 (1.02–1.18)**
(CCI = 0)	CCI > 2	**2.08 (1.94–2.24)**	**1.99 (1.85–2.14)**	**1.20 (1.09–1.32)**	**1.15 (1.05–1.27)**
Stage	B	**1.87 (1.45–2.41)**	**2.05 (1.59–2.65)**	**3.01 (1.93–4.71)**	**3.21 (2.07–4.99)**
(stage A)	C	**3.21 (2.53–4.07)**	**3.99 (3.12–5.09)**	**7.36 (4.52–10.60)**	**8.76 (5.71–13.44)**
	D	**12.33 (9.76–15.56)**	**13.26(10.43–16.85)**	**30.95 (20.23–47.35)**	**34.23 (22.34–52.44)**
	Unknown	**1.77 (1.39–2.26)**	**1.47 (1.15–1.87)**	**3.38 (2.18–5.23)**	**2.80 (1.82–4.31)**
Primary site	Left colon	1.01 (0.91–1.11)	1.04 (0.97–1.11)	**1.11 (1.02–1.21)**	1.05 (0.96–1.14)
(right colon)	Rectum	0.91 (0.83–1.01)	0.96 (0.89–1.03)	**1.18 (1.09–1.27)**	0.98 (0.90–1.08)
Surgery (non-receipt)		–	**0.48 (0.44–0.52)**	–	**0.52 (0.47–0.57)**
Chemotherapy (non-receipt)		–	**0.73 (0.67–0.81)**	–	**0.63 (0.56–0.70)**
Radiotherapy (non-receipt)		–	0.93 (0.83–1.04)	–	0.90 (0.80–1.02)

^*^Significant results are shown in bold, ^†^adjusted for age, CCI, stage, primary subsite, grade, socioeconomic and remoteness status, diagnostic period, sex,^‡^ addition of treatments in addition to previous adjustments. Time varying covariates for stage, grade, surgery, chemotherapy, and radiotherapy, HR, Hazard ratio; SHR, Sub-hazard ratio; CI, confidence interval; CCI, Charlson comorbidity index.

### 3.3. Mortality measures after inclusion of treatments

Recipients of chemotherapy and surgery had significantly lower mortalities. Radiotherapy was also associated with lower mortality, but it wasn't statistically significant. There was not a great difference between the models after adjustment for treatment modalities (neither for all-cause nor CRC-specific mortality). However, the associations for age and comorbidity became weaker after addition of treatment modalities to the models (Table 3). Associations left colon and rectal cancer with CRC-specific mortality was no longer significant after adjusting for treatments.

### 3.4. How stage, subsite and comorbidity affect the association of age with mortality

#### 3.4.1. Stage

Younger age was associated with lower all-cause and CRC-specific mortality for all stages compared with >80 years as the reference (Supplementary Table S1). However, in all-cause mortality, these associations were stronger for earlier stages (A, B) than advanced stages (C, D); as people between 51 and 80 were less likely to experience all-cause mortality in comparison to the 80+ age group when they had earlier stages (e.g., for the 70–79 age group: for stage A, B 95% CI = 0.25–0.36 vs. for stage C 95% CI = 0.60–0.83). This difference was not observed for CRC-specific mortality.

The interaction term analysis between age group and stage showed that the association of age with all-cause mortality is significantly dependent on the stage of the disease, with people under 80 years having a higher risk of dying relative to older people when they have stages C and D (Supplementary Table S2). This correlation is also observed to a lesser extent for CRC-specific mortality. This shows that aging has a greater influence on the association of early-stage CRC with mortality than advanced-stage CRC.

#### 3.4.2. Subsite

Younger age was associated with lower all-cause and CRC-specific mortality in both colon and rectal cancer (Supplementary Table S3). This significant associations were not different across different site (i.e., right, left colon and rectum).

Supplementary Table S4 shows that the association of age group with mortality measures (especially CRC-specific) was “statistically significantly” different by subsite, with people with rectal cancer and younger than 80 years being less likely to have both all-cause and CRC-specific mortality (compared to the reference group) than patients with right colon cancer [e.g., for age group 71–80 and rectal cancer SHR = 0.69 (0.55–0.086)]. No difference of association of age with mortality was observed between right and left colon.

#### 3.4.3. Comorbidity

Supplementary Table S5 indicates that younger age was significantly associated with lower all-cause mortality regardless of the CCI status. For CRC- specific mortality, the significance of the associations only existed for those with CCI ≤ 2, and those with CCI > 2 had no significant association of age with CRC-specific mortality.

Interaction terms indicated that the association between age group and both mortality measures did not vary to a “statistically significant” extent with level of comorbidity, except for all-cause mortality in 71–80 year when those older than 80 are significantly more likely to experience all-cause mortality when they have CCI > 2 (Supplementary Table S6).

### 3.5. Specific comorbidities

**Figure 2:**
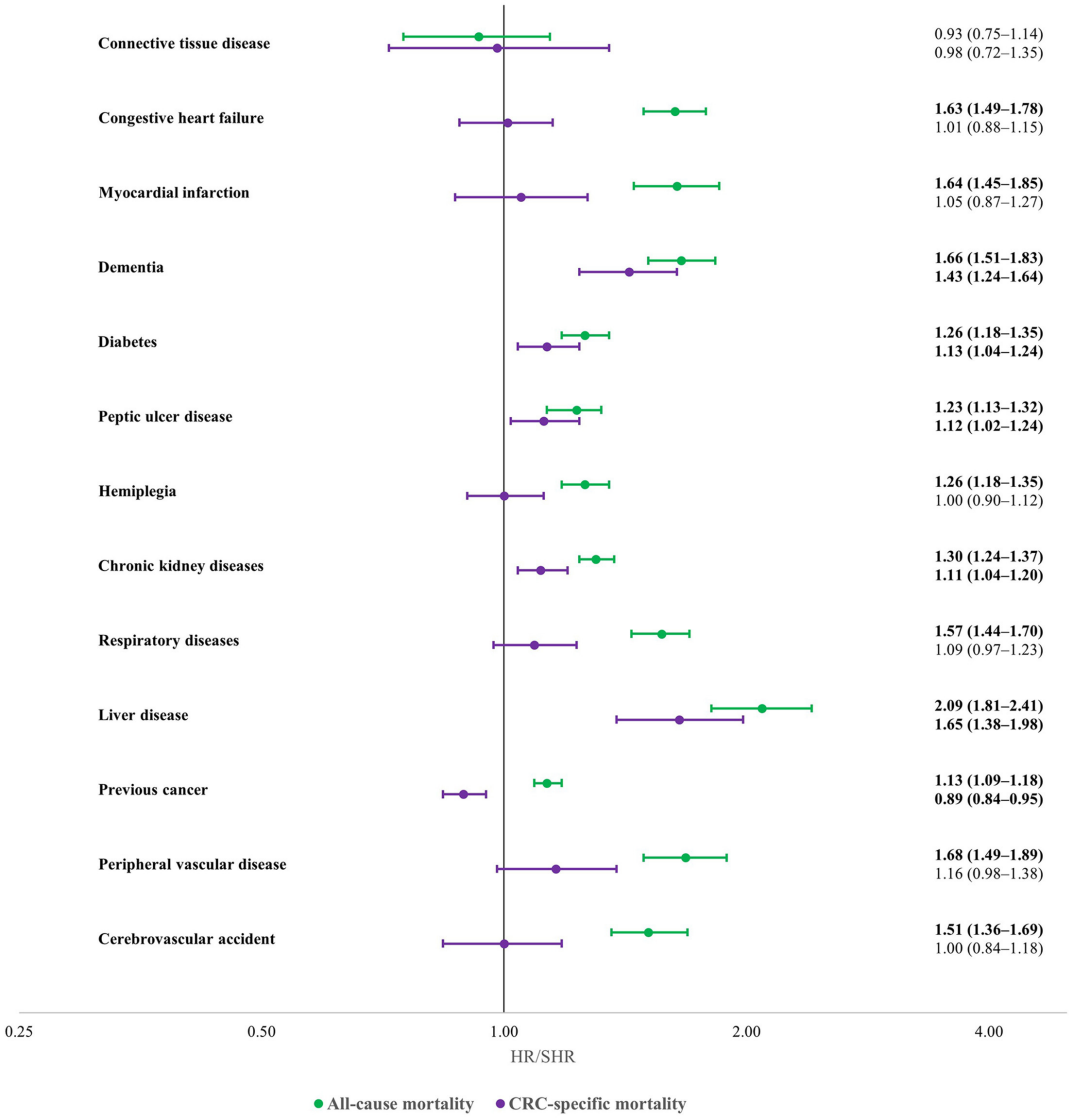
Associations of specific comorbidities with all-cause (green) and CRC-specific (purple) mortality in CRC cases (multivariable regression): South Australia, 2004–2013 diagnose, date of censoring December 31, 2014 adjusted for age, stage, primary subsite, grade, socioeconomic and remoteness status, diagnostic period, sex. Statistically significant results are shown with bold font. All-cause mortality outcomes are reported as hazard ratio (HR), and CRC-specific mortality outcomes are reported as sub-distribution hazard ratio (SHR).

Figure 2 indicates associations of individual comorbidities in CCI with all-cause and CRC-specific mortality. All the listed comorbidities except for connective tissue disease (HR = 0.93, 95% CI = 0.75–1.14) were associated with all-cause mortality. Dementia, diabetes, Peptic ulcer disease, chronic kidney disease, and liver diseases were the only comorbidities, however, that were associated with CRC-specific mortality.

## 4. Discussion

Several studies have already shown advanced age and comorbidity to be associated with all-cause and CRC-specific mortality (21, 22). It's previously found that age and primary subsite are associated with stage of CRC. It is therefore relevant to consider the influences of these factors on associations of age with survival. Kaplan-Meier analyses indicated that the CRC-specific survival was quantitatively close to survival from all causes in the first 5 years after CRC diagnosis. However, within 5 to 10 years after diagnosis the difference of the two survivals tends to increase especially among older age groups. This shows that most cases die due to CRC in the first years of the diagnosis, but non-CRC mortality is more likely to happen after the first years of CRC diagnosis.

Our results were consistent with those from earlier studies indicating that age and comorbidity were stronger determinants of all-cause than CRC-specific mortality (5, 8, 23). An earlier study found patients with the comorbidities were more likely to die of circulatory diseases and other cancers than of CRC (24). In total, these findings underscore the importance of addressing chronic comorbidity in CRC care, particularly among older patients. Studies of CRC mortality for different subsites have conflicting results based on the methodology and stage of the cancer (25–27). Our results showed no difference of overall survival based on the subsite of CRC. CRC-specific mortality was higher for left colon and rectal cancer before adjusting for treatment, which indicates difference in this mortality mostly stems from different treatment pattern based on subsite.

Addition of treatment modalities to our predictive models slightly weakened the associations of age and comorbidity with mortality. Associations of stage with mortality however experienced an increase in significance. The data indicated that treatment modalities were potential confounders of associations of stage with mortality. Surgery had the strongest association with mortality measures compared with chemotherapy and radiotherapy.

The strength of associations of age with CRC mortality measures reduced with increasingly advanced stage and for unknown stage. That is, for advanced stages, the association of younger age with better prognosis was weaker. An earlier study found the association of advanced age with all-cause mortality to be stronger for stage I and II than stage III, although an equivalent gradient was not observed for CRC-specific survival (8). The weaker association of age with mortality for advanced stages may reflect the higher mortality experienced by younger people with advanced stage, which acted to increase likelihood of death to a level more akin to that seen in older people. As a result, there was a clearer aging effect on mortality for early-stage than late-stage patients.

The association of age with mortality also differed by cancer subsite, being stronger for rectal than right colon cancer. In other words, the association of advanced age with increased likelihood of mortality appeared greater for rectal than right colon cancers. A possible reason could be differences in treatment, in that rectal cancer patients have more chemotherapy, both adjuvant and neoadjuvant, and often were treated by radiotherapy. Such treatments are more likely to cause toxicity in older patients, leading to poorer survival in these people (28–30).

We are not aware of studies that previously investigated variations in associations of age with mortality across different levels of comorbidity. Our analyses indicated that older people were more likely to experience CRC-specific death, and deaths from all causes of death, irrespective of comorbidity status except for all-cause mortality in those older than 70. In other words, effects of aging on mortality appeared to be largely unchanged irrespective of comorbidity level which means advanced age is almost as a great contributor to mortality for people with no comorbidity that it is for those with higher levels of comorbidity. However, in case of inclusion of factors that are confounders of comorbidity (e.g., performance status, treatment intensity or frailty), we might have found more significant difference in association of age with mortality across different comorbidity levels.

Other studies have shown that most individual comorbidities are associated with increased all-cause mortality in CRC patients (9, 31). Unlike all-cause mortality, however, we found few specific comorbidities to be associated with CRC-specific mortality. Kidney disease was associated with increased CRC-specific mortality as found by other researchers (5). We also found peptic ulcer disease and dementia to be associated with CRC-specific mortality, which were more novel findings. Patients with kidney, liver and gastric diseases generally are given reduced doses of chemotherapy which may predispose to an increased likelihood of CRC-specific mortality (32, 33). The association of peptic ulcer disease increased CRC-specific mortality is more difficult to explain, especially as this disease is commonly controlled by diet and medications. In this study, however, the cases were hospitalized and potentially of greater severity with increased gastrointestinal bleeding (15). People with previous cancers were less likely to have CRC-specific mortality. The reasons are uncertain but might include increased likelihood of attributions of deaths to other cancers. Another potential explanation is that these people might have received several chemotherapy treatments for both their cancers. Evidence already has been reported that people with complications of chemotherapy (e.g., hematological and gastric toxicities) are less likely to have a CRC-death, with most dying of other causes (24).

### 4.1. Implications and further research

It is evident from registry data reported by the Australian Institute of Health and Welfare that survival of CRC patient declines precipitously from the age of 80 years (1). It will be important in future research to explore reasons, including the potential for lower referral rates to multidisciplinary teams and lower participation in clinical trials.

We found that associations of age with mortality is not significantly influenced by the level of comorbidity. This means that younger people of any comorbidity status had equally a better prognosis than their counterparts older than 79. These results showed that the poor prognosis of older people which is believed to be partly due to comorbidities in this population (34), similarly exists for people without comorbidities. The relationship may be complicated, however, and further research is needed to explore causes other than comorbidity for the poorer prognosis of older CRC patients (e.g., frailty). Another possible explanation for poorer outcomes in old patients is variations in treatment complications, such as toxicities (e.g., neurologic, hematological etc.), perforations, and infections. We lacked the range and quality of data to explore these possibilities.

Even though rectal cancer has lower all-cause mortality, the cancer-specific mortality is significantly higher in our analysis. Some studies have reported that unlike colon cancer, rectal cancer mortality has an increasing trend in some countries including Australia, Canada and the United States and needs to be further investigated (35, 36). It is also shown that even though older people are experiencing higher mortality and the number of CRC deaths is going to rise because of the growing incidence rates; age groups younger than 50 are the only ones with an increasing projection of CRC mortality and more studies are needed in this area (37, 38).

Unlike all-cause mortality, only some comorbidities appear to change the CRC-specific mortality of patients. These comorbidities may affect treatment options and could include lowering doses of chemotherapy or delaying surgery. Studies are needed into mechanisms whereby co-existing conditions can alter the CRC-specific mortality such as by interacting with the pathogenesis of the disease or treatment.

Our results showed the groups in population that are more vulnerable to aging. Those with early stage are more likely to experience mortality as they age compared with their counterparts with advanced stage. Likewise, aging has a greater influence on rectal cancer mortality than colon cancer. This shows that more emphasis should be placed on early detection of CRC in these groups. Conversely, mortality likelihood of younger people with advanced stage or colon cancer is significantly closer to their older counterparts when being compared to those with early stage or rectal cancer respectively, which indicates the necessity of finding better treatment strategies for these groups.

### 4.2. Strengths and limitations

A strength of this study was long-term follow-up through linkages to the BDM registry. This would have minimized the chance of under-detection of deaths. The study also achieved broad coverage of South Australian CRC population. Many analyses in this study, including the subgroup analyses by subsite and comorbidity status, and analyses of individual comorbidities, were novel and pointed to factors that may predispose to poorer prognosis in older CRC patients. These factors need to inform strategic priorities for reducing CRC mortality.

A limitation of the study is that the data used for this research were not originally collected for research purposes which caused some patients to have different follow-up times based on the time of diagnosis and a proportion of the cases had unknown stages. Some important variables could also improve our results such as frailty indices or treatment type and intensity. Obtaining health administrative data in Australia requires extensive scrutiny of applications through research and ethics committees which is time-consuming and causes delays in reporting results. However, the main focus of this study (i.e., the correlation of age and comorbidity with CRC mortality) may not have been unduly affected by this lack of recency. CCI which was the measuring tool for comorbidity level which has limitations such as not having all main comorbidities (16, 39), but it provides a general score which was generally shown to be associated with mortality (39, 40).

## 5. Conclusion

This study confirms that advanced age and comorbidities are associated with all-cause and CRC-specific mortality. They are more likely, however, to increase the probability of all-cause mortality than CRC-specific mortality. Treatments were negatively associated with mortality measures, and they also moderated the association of advanced age, comorbidity and subsite with mortality. The positive association of mortality with age was observed to be mediated by stage and subsite. However, level of comorbidity was not found to significantly alter the association of age with mortality. We found only a limited number of comorbidities to be associated with CRC-specific mortality. This is a novel finding that requires confirmation in independent studies. Increased attention should be given to studies into the factors responsible for poorer prognosis of older patients with colorectal cancer, including studies of customized design and data collection to obtain the best possible evidence.

## Data availability statement

The raw data supporting the conclusions of this article are protected by the AIHW and cannot be made readily available. Any queries can be directed to the corresponding author(s).

## Ethics statement

The studies involving human participants were reviewed and approved by SA Health (HREC/16/SAH/6/AM04) and University of South Australia (EO2016/4/317). Written informed consent for participation was not required for this study in accordance with the national legislation and the institutional requirements.

## Author contributions

KG drafted the manuscript and performed statistical analysis. EB supervised the statistical methodology and study design. AV helped the study in terms of therapeutical methods for CRC and review of the literature. DR and his team obtained the study data and he supervised the whole project. All authors contributed to the article and approved the submitted version.
