# Supplementary material for: Associations of advanced age with comorbidity, stage and primary subsite as contributors to mortality from colorectal cancer
Source: Front Public Health. 2023 Apr 6;11:1101771. doi: 10.3389/fpubh.2023.1101771 (PMC10116414; doi:10.3389/fpubh.2023.1101771)
Supplement: Supplementary file 1 [file Data_Sheet_1.pdf]

Table 1S*. Associations of age with mortality measures by stage (ACPS) (multivariable regression): South Australia, 2004-2013 diagnose, date of censoring December 31, 2014**

Variable (reference)		Hazard ratio (95% confidence interval) for all-cause mortality<br>Stages A, B<br>N=3029	Stage C<br>N=2850	Stage D<br>N=2419	Unknown stage<br>N=3020
Age group (>80)	≤50	**0.10 (0.06-0.18)**	**0.48 (0.36-0.64)**	**0.64 (0.53-0.78)**	**0.13 (0.09-0.18)**
	51-60	**0.13 (0.10-0.17)**	**0.51 (0.41-0.63)**	**0.74 (0.63-0.88)**	**0.38 (0.29-0.49)**
	61-70	**0.16 (0.13-0.21)**	**0.56 (0.46-0.68)**	**0.76 (0.65-0.88)**	**0.44 (0.36-0.53)**
	71-80	**0.30 (0.25-0.36)**	**0.71 (0.60-0.83)**	**0.83 (0.79-0.94)**	**0.55 (0.47-0.64)**
Variable (reference)		Sub-hazard ratio (95% confidence interval) for CRC-specific mortality<br>Stages A, B<br>N=3029	Stage C<br>N=2850	Stage D<br>N=2419	Unknown stage<br>N=3020
Age group (>80)	≤50	**0.27 (0.14-0.50)**	**0.66 (0.48-0.91)**	**0.73 (0.59-0.90)**	**0.23 (0.15-0.33)**
	51-60	**0.28 (0.19-0.41)**	0.82 (0.64-1.05)	0.91 (0.76-1.09)	**0.68 (0.50-0.93)**
	61-70	**0.30 (0.22-0.42)**	**0.76 (0.60-0.96)**	0.91 (0.77-1.08)	**0.75 (0.59-0.96)**
	71-80	**0.44 (0.32-0.59)**	0.82 (0.67-1.01)	0.95 (0.83-1.10)	**0.74 (0.60-0.91)**

"Statistically significant" results shown in bold. Model additionally adjusted for primary subsite, comorbidity level, grade, socioeconomic status, remoteness, diagnostic period, sex and treatments, ACPS: Australian Clinico-pathological Stage

Table 2S. *Association of interaction terms for age and stage (ACPS) with mortality measures (multivariable regression): South Australia, 2004-2013 diagnose, date of censoring December 31, 2014*

Reference	Variables	All-cause mortality HR (95% confidence interval)	CRC-specific mortality SHR (95% confidence interval)
>80 year # stage A, B	≤50 year # stage C	**3.24 (1.80-5.81)**	1.28 (0.65-2.54)
	≤50 year # stage D	**5.19 (2.96-8.99)**	1.68 (0.87-3.21)
	≤50 year # unknown	1.31 (0.71-2.42)	1.14 (0.57-2.30)
	51-60 year # Stage C	**2.66 (1.92-3.67)**	1.46 (0.97-2.20)
	51-60 year # Stage D	**4.48 (3.30-6.06)**	**1.91 (1.29-2.83)**
	51-60 year # unknown	**3.05 (2.15-4.32)**	**2.86 (1.91-4.29)**
	61-70 year # stage C	**2.30 (1.77-2.98)**	1.31 (0.91-1.88)
	61-70 year # stage D	**3.57 (2.81-4.54)**	**1.86 (1.32-2.61)**
	61-70 year # unknown	**2.64 (2.01-3.46)**	**2.18 (1.52-3.11)**
	71-80 year # stage C	**1.83 (1.46-2.29)**	1.20 (0.86-1.69)
	71-80 year # stage D	**2.24 (1.82-2.76)**	**1.57 (1.14-2.17)**
	71-80 year # unknown	**1.83 (1.46-2.28)**	**1.62 (1.16-2.26)**

"Statistically significant" results shown in bold. Model additionally adjusted for primary subsite, comorbidity level, grade, socioeconomic status, remoteness, diagnostic period, sex and treatments, ACPS: Australian Clinico-pathological Stage

Table 3S*. Association of age with mortality measures by subsite (multivariable regression): South Australia, 2004-2013 diagnose, date of censoring December 31, 2014*

Variable (reference)		Hazard ratio (95% confidence interval) for all-cause mortality		
		Right colon N=4525	Left colon N=3096	Rectum N=3697
Age group (>80)	≤50	**0.32 (0.25-0.41)**	**0.38 (0.29-0.49)**	**031 (0.25-0.39)**
	51-60	**0.47 (0.39-0.56)**	**0.42 (0.34-0.51)**	**0.41 (0.35-0.49)**
	61-70	**0.46 (0.40-0.53)**	**0.48 (0.40-0.56)**	**0.47 (0.40-0.55)**
	71-80	**0.64 (0.57-0.71)**	**0.60 (0.52-0.69)**	**0.57 (0.50-0.66)**
Variable (reference)		Sub-hazard ratio (95% confidence interval) for CRC-specific mortality		
		Right colon N=4525	Left colon N=3096	Rectum N=3697
Age group (>80)	≤50	**0.54 (0.41-0.71)**	**0.53 (0.40-0.69)**	**0.43 (0.33-0.55)**
	51-60	**0.78 (0.63-0.96)**	**0.70 (0.56-0.89)**	**0.64 (0.52-0.79)**
	61-70	**0.77 (0.64-0.91)**	**0.71 (0.58-0.87)**	**0.63 (0.52-0.77)**
	71-80	**0.84 (0.72-0.98)**	**0.76 (0.63-0.92)**	**0.70 (0.58-0.85)**

"Statistically significant" results shown in bold. Model additionally adjusted for stage, primary subsite, comorbidity level, grade, socioeconomic status, remoteness, diagnostic period, sex and treatments.

Table 4S. *Association of interaction terms for age and subsite with mortality measures (multivariable regression): South Australia, 2004-2013 diagnose, date of censoring December 31, 2014*

Reference	Variables	All-cause mortality HR (95% confidence interval)	CRC-specific mortality SHR (95% confidence interval)
>80 year # Right colon	≤50 year # left colon	1.08 (0.78-1.50)	0.89 (0.62-1.26)
	≤50 year # Rectum	0.81 (0.60-1.10)	**0.59 (0.41-0.83)**
	51-60 year # left colon	0.91 (0.71-1.16)	0.85 (0.64-1.11)
	51-60 year # Rectum	**0.76 (0.61-0.95)**	**0.62 (0.47-0.80)**
	61-70 year# left colon	1.03 (0.84-1.25)	0.86 (0.68-1.09)
	61-70 year # Rectum	0.90 (0.74-1.09)	**0.64 (0.51-0.82)**
	71-80 year # left colon	0.92 (0.78-1.09)	0.85 (0.68-1.07)
	71-80 year # Rectum	**0.81 (0.69-0.96)**	**0.69 (0.55-0.86)**

"Statistically significant" results shown in bold. Model additionally adjusted for stage, comorbidity level, primary subsite, grade, socioeconomic status, remoteness, diagnostic period, sex and treatments.

Table 5S. *Association of age with mortality measures by CCI status (multivariable regression): South Australia, 2004-2013 diagnose, date of censoring December 31, 2014*

Reference: (>80 year)	Hazard ratio (95% confidence interval) for all-cause mortality		
	CCI=0<br>N=5900 cases	CCI=1,2<br>N=3497 cases	CCI>2<br>N=1921 cases
≤50	**0.29 (0.24-0.34)**	**0.33 (0.25-0.43)**	**0.49 (0.31-0.77)**
51-60	**0.40 (0.34-0.46)**	**0.43 (0.35-0.51)**	**0.54 (0.41-0.70)**
61-70	**0.42 (0.36-0.48)**	**0.49 (0.42-0.57)**	**0.57 (0.47-0.68)**
71-80	**0.55 (0.48-0.62)**	**0.60 (0.53-0.68)**	**0.76 (0.67-0.87)**
Reference: (>80 year)	Sub-hazard ratio (95% confidence interval) for CRC specific mortality		
	CCI=0<br>N=5900 cases	CCI=1,2<br>N=3497 cases	CCI>2<br>N=1921 cases
≤50	**0.45 (0.37-0.55)**	**0.50 (0.37-0.67)**	0.68 (0.44-1.06)
51-60	**0.64 (0.54-0.77)**	**0.76 (0.61-0.94)**	0.81 (0.65-1.16)
61-70	**0.67 (0.57-0.80)**	**0.72 (0.60-0.87)**	0.78 (0.67-1.07)
71-80	**0.75 (0.63-0.88)**	**0.78 (0.66-0.91)**	0.90 (0.75-1.08)

"Statistically significant" results shown in bold. Model additionally adjusted for stage, primary subsite, grade, socioeconomic status, remoteness, diagnostic period, sex and treatments. CCI: Charlson Comorbidity Index

Table 6S. *Association of the interaction terms for age and CCI status with mortality measures (multivariable regression): South Australia, 2004-2013 diagnose, date of censoring December 31, 2014*

Reference	Variables	All-cause mortality HR (95% confidence interval)	CRC-specific mortality SHR (95% confidence interval)
>80 year # CCI=0	≤50 year # CCI=1,2	0.89 (0.65-1.20)	0.92 (0.65-1.29)
	≤50 year # CCI>2	1.20 (0.75-1.94)	1.27 (0.78-2.05)
	51-60 year # CCI=1,2	0.91 (0.73-1.12)	1.05 (0.82-1.34)
	51-60 year # CCI>2	1.06 (0.80-1.41)	1.23 (0.89-1.70)
	61-70 year # CCI=1,2	1.03 (0.86-1.23)	0.96 (0.77-1.20)
	61-70 year # CCI>2	1.17 (0.95-1.43)	1.19 (0.91-1.54)
	71-80 year # CCI=1,2	1.00 (0.85-1.18)	0.96 (0.78-1.18)
	71-80 year # CCI>2	**1.25 (1.06-1.49)**	1.16 (0.91-1.47)

Model additionally adjusted for stage, primary subsite, grade, socioeconomic status, remoteness, diagnostic period, sex and treatments, CCI: Charlson Comorbidity Index

***Figure 1S**. Kaplan-Meier curves for all-cause and CRC-specific survival by age: South Australia, 2004-2013 diagnose, date of censoring December 31, 2014*

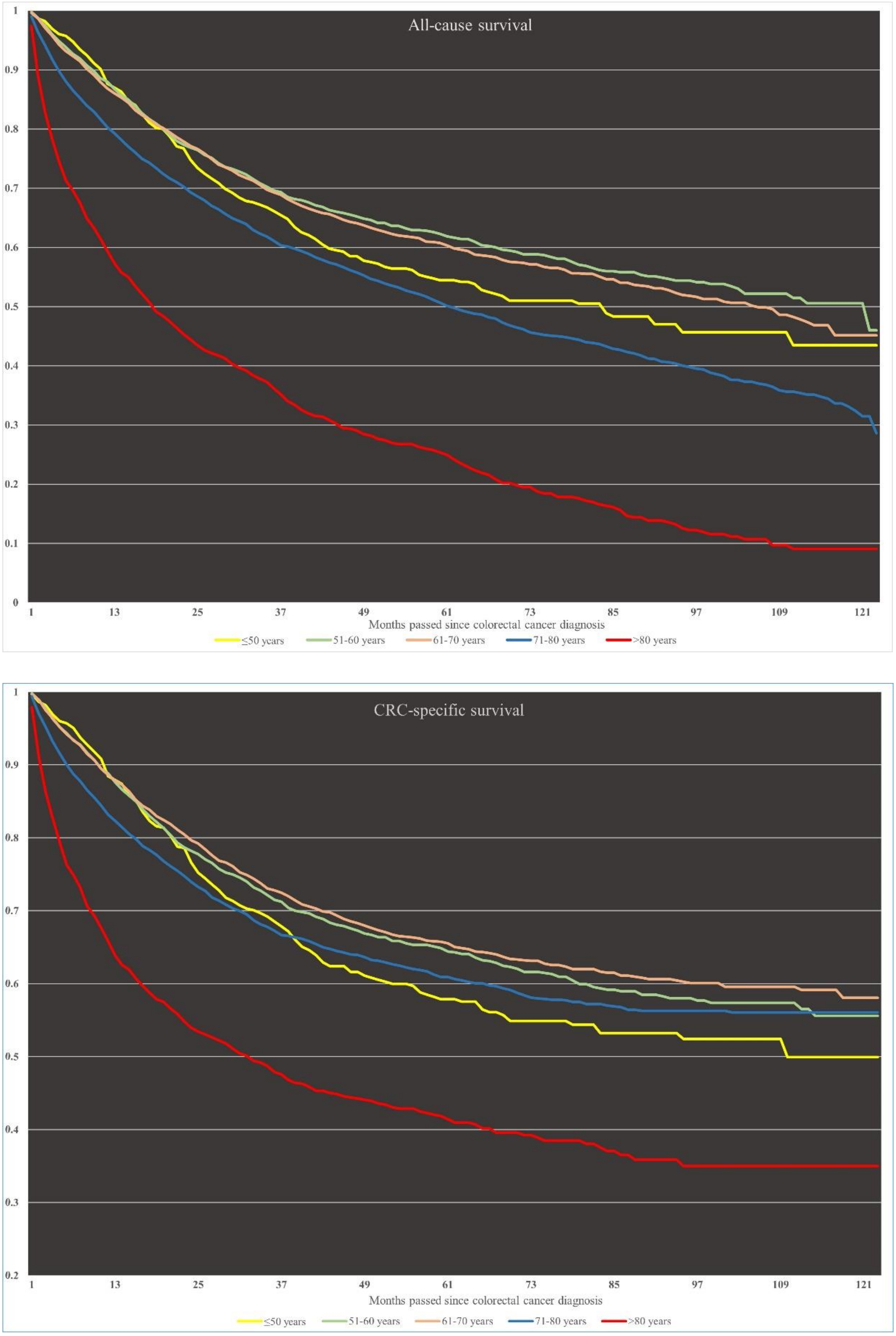

CRC: colorectal cancer
